# Real-time earthquake monitoring using a search engine method

**DOI:** 10.1038/ncomms6664

**Published:** 2014-12-04

**Authors:** Jie Zhang, Haijiang Zhang, Enhong Chen, Yi Zheng, Wenhuan Kuang, Xiong Zhang

**Affiliations:** 1 Laboratory of Seismology and Physics of Earth’s Interior, School of Earth and Space Sciences, University of Science and Technology of China, Hefei, Anhui 230026, P.R. China; 2 School of Computer Science and Technology, University of Science and Technology of China, Hefei, Anhui 230027, P.R. China

## Abstract

When an earthquake occurs, seismologists want to use recorded seismograms to infer its location, magnitude and source-focal mechanism as quickly as possible. If such information could be determined immediately, timely evacuations and emergency actions could be undertaken to mitigate earthquake damage. Current advanced methods can report the initial location and magnitude of an earthquake within a few seconds, but estimating the source-focal mechanism may require minutes to hours. Here we present an earthquake search engine, similar to a web search engine, that we developed by applying a computer fast search method to a large seismogram database to find waveforms that best fit the input data. Our method is several thousand times faster than an exact search. For an *M*_w_ 5.9 earthquake on 8 March 2012 in Xinjiang, China, the search engine can infer the earthquake’s parameters in <1 s after receiving the long-period surface wave data.

## Abstract (web summary)

Reporting earthquakes, including location and focal mechanism, in real time is a challenge. Here, the authors present an approach similar to a web search engine, estimating earthquake parameters by searching a large database within a second, which will potentially enable early warning systems.

## Graphical abstract

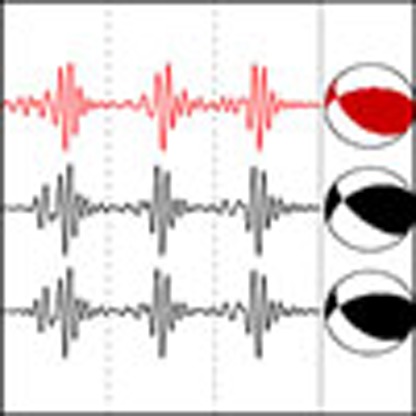

Reporting earthquakes in real time has long been a significant research effort in the seismological community^1^,^2^,^3^. In recent years, such efforts have been further refined towards developing earthquake early warning systems that can issue warnings to the public within a few seconds to slightly >1 min after the event occurs^4^. Several earthquake early warning systems have been implemented around the world, including REIS in Japan^5^, SAS in Mexico^6^, VSN in Taiwan^7^ and IERREWS in Turkey^8^. In the United States, significant research efforts have been put into the development of the ElarmS early warning system in California, which has been shown to be effective in offline tests but has not been fully implemented yet^4^. Seismologists have developed robust algorithms to estimate the source information from earthquakes automatically^9^,^10^. For example, in Japan, the REIS system allows the estimation of the earthquake’s location and magnitude within 5 s after the P-wave arrival by using data from a dense monitoring network^5^. However, it still takes several minutes or more to derive the source-focal mechanism, even with the recently published new methods that invert moment tensors with Green’s functions calculated in advance for potential earthquake locations over a grid^11^,^12^,^13^. Similar efforts using GPS data can also determine the centroid moment tensors of large earthquakes in minutes^14^,^15^.

In addition to the location and magnitude, it is important to derive the source-focal mechanism for earthquakes in real time. For example, tsunami prediction requires complete source parameters, including source depth, magnitude, slip and orientation of the fault^16^. A shallow earthquake with a seismic moment magnitude of *M*_w_ 7.7 occurred off the west coast of Sumatra on 25 October 2010, generating a local tsunami that reached a height of 3 m and hit the islands in minutes. More than 400 fatalities were reported^17^. A focal mechanism study revealed that this earthquake had a thrust-faulting mechanism, causing significant seawater movement. By contrast, a large Indian Ocean earthquake on 11 April 2012, with a magnitude of *M*_w_ 8.6 and followed ~2 h later by a large (*M*_w_ 8.2) aftershock, did not cause a tsunami, although warnings were issued across the Indian Ocean. The focal mechanism solutions suggest that both earthquakes were caused by strike-slip motion^18^; thus, the movement displaced relatively little seawater and was less likely to cause a tsunami. The real-time estimation of source-focal mechanism is also important for monitoring fault activities. For example, Bouchon *et al.*^19^ analysed the extended nucleation of the 1999 *M*_w_ 7.6 Izmit earthquake and found a sequence of foreshocks whose source-focal mechanisms indicate similar fault slips before accelerating to dynamic rupture. Obtaining the focal mechanisms of earthquake swarms in real time may help characterise fault activities. Such information could immediately attract our attention to the seismically active area and delineate the fault movement in real time.

The challenge lies in the automatic and rapid estimation of the earthquake source mechanism in a few seconds after receiving the seismic data from a few stations. We develop an image-based earthquake search engine, similar to web search engines, to estimate earthquake parameters within 1 s by searching for similar seismograms from a large database on a single AMD Opteron processor 6136. Significant advances in computer search technology have helped the search industry to retrieve words, images, videos and voices from Internet-sized data sets^20^,^21^,^22^,^23^,^24^,^25^,^26^,^27^,^28^,^29^,^30^,^31^,^32^. Similar to voice recording or a one-dimensional (1D) image, a seismogram is a graph record of the ground motion at a recording station as a function of time. It contains information about both the earthquake source and the earth medium through which the waves propagated. By assuming that the earth velocity model is known, we apply a forward modelling approach to build a database of waveforms for scenario earthquakes over possible source mechanisms and locations on a discretised grid. Our objective is to find the best matches to any new earthquake record from the database. This approach is fully automatic and requires no parameter input or human interference. Therefore, it could be applied for routinely reporting earthquake parameters.

We test our earthquake search engine using three real earthquakes in a test site in Xinjiang, China. An area 5° by 5° in latitude and longitude is selected to create a fast-search database. The search results can be obtained by the search engine in <1 s in all three cases after receiving the long-period surface wave data. For an event within the database coverage area, the determined earthquake source parameters are sufficiently accurate. If an event is outside the database coverage area, or multiple events are closely spaced in time, the search engine can automatically invalidate the results using a predefined cross-correlation threshold.

## Results

### Search engine database for the Xinjiang test site

**Figure 1:**
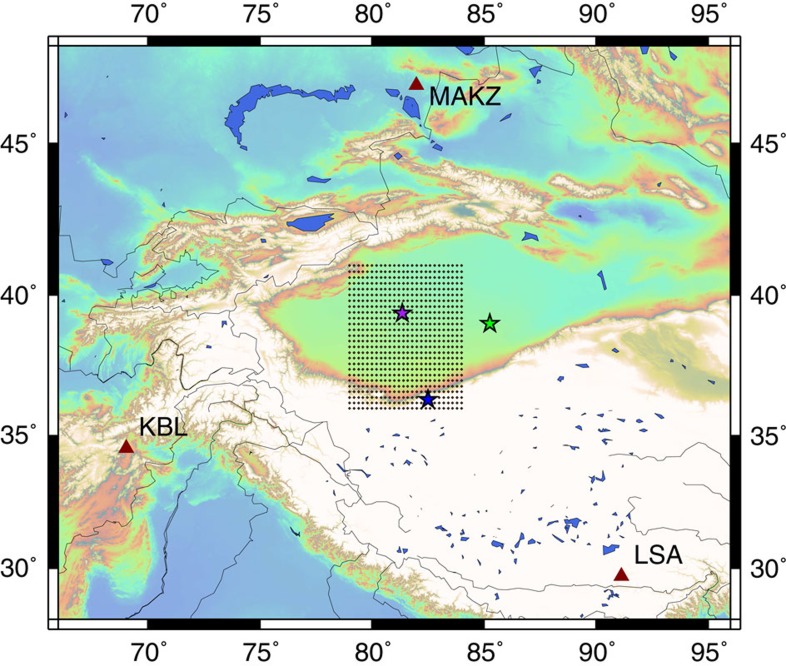
The locations of the seismic stations and the virtual test site. A large synthetic data set from virtual sources in the area of three seismic stations denoted by dark red triangles (MAKZ—46.8°N, 82.0°E in Makanchi, Kazakhstan; KBL—34.5°N, 69.0°E in Kabul, Afghanistan; LSA—29.7°N, 91.1°E in Lhasa, China) is generated to create a fast search database and three earthquakes denoted by stars (locations from the United States Geological Survey (USGS)—NEIC) are used to test different situations. The purple star denotes an earthquake of a magnitude of *M*_w_ 5.9 that occurred on 8 March 2012; the blue star denotes an earthquake of a magnitude of *M*_w_ 5.3 on 15 September 2011; and the green star denotes an earthquake of a magnitude of *M*_w_ 5.3 on 30 April 2014.

Figure 1 shows a test area of 5° by 5° in Southern Xinjiang, China, where three permanent seismic stations (dark red triangles) can record earthquakes from the area within a range of ~5° to 15°. These stations include MAKZ (46.8°N, 82.0°E in Makanchi, Kazakhstan), KBL (34.5°N, 69.0°E in Kabul, Afghanistan) and LSA (29.7°N, 91.1°E in Lhasa, China). From 1 January 2000 to present, 51 earthquakes with magnitudes >*M*_w_ 4.0 occurred in the test site according to the United States Geological Survey—National Earthquake Information Centre (NEIC). In this study, we focus on the real-time analysis of small-to-moderate earthquakes to determine their hypocentres and double-couple focal mechanisms in this area. Our approach includes the following steps: (1) the construction of a synthetic database with Green’s functions calculated using a laterally homogeneous medium for ‘virtual sources’ on discretised grid nodes in the study region and convolved with all possible double-couple source solutions; (2) reduction of the number of time samples in the database by principal components analysis; (3) use of the fast search algorithm developed in the internet industry to quickly find the best-matching ‘virtual sources;’ and (4) validation of the solutions and quantification of the resolution of the search results.

To design an earthquake search engine specifically for the monitoring of this test site, we create a database consisting of a large number of synthetic seismograms corresponding to every virtual source point in the 3D grid within the test site. Our three-component seismogram calculation applies the elastic wave modelling of a point earthquake source in a multilayered half-space using the Thompson–Haskell propagator matrix technique^33^. In this study, we primarily focus on the shallow earthquakes within the test site recorded at distances of 5° to 15°. We tested the PREM earth model^34^ by modelling a few historic events for the area of interest. It is reasonable to use this model to simulate the long-period seismic wavefield (0.01–0.05 Hz) propagating in the area. We also tested the search engine with real earthquakes inside and outside the test site, as well as with a ‘hypothesised’ double-event earthquake constructed from two real earthquakes.

As shown in Fig. 1, the test area is gridded in 0.2° intervals in latitude from 36°N to 41°N and in longitude from 79°E to 84°E. The depth grid is from 5 to 60 km, with an interval of 5 km. Thus, there are 8,112 virtual source points in the 3D grid. The source-focal mechanism at each grid point is discretised as follows: strike ranging from 10° to 350° with an interval of 20°, dip ranging from 5° to 80° with an interval of 15° and rake ranging from −170° to 170° with an interval of 20°. This leads to a total of 1,944 different events at each virtual source point. Therefore, we should generate three-component seismograms for 15,789,168 (8,122 × 1,944) earthquakes in the 3D grid for every seismic station, that is, a total of 47,367,504 three-component seismograms for the three seismic stations. To create the search database, we merge the three-component seismograms from all the three seismic stations and produce a long super-trace for each virtual earthquake. Hence, there are a total of 15,789,168 super-traces in the search database.

For any virtual source position, the greatest computational effort is the calculation of the Green’s function between source and receiver before convolving it with a focal solution. For a 1D Earth model, there should be nine sets of Green’s functions and eight of them contribute to the calculation of the three-component seismogram for a double-couple source^13^,^33^. Fortunately, the Green’s function is independent of the source-focal mechanism; thus, we only need to calculate one set of Green’s function for each grid point.

**Figure 2:**
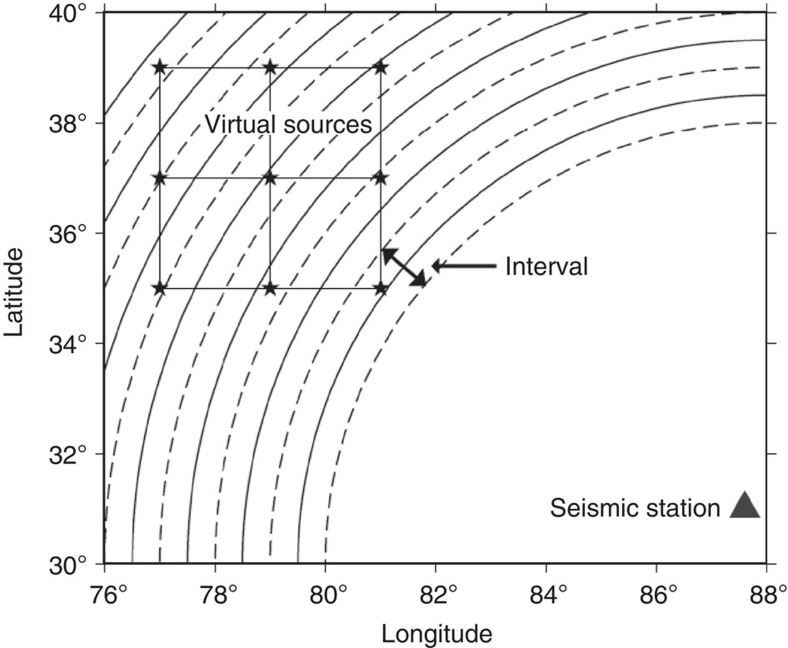
An interval approach for preparing the search database. From a seismic station, solid circles with a constant interval are defined. A synthetic Green’s function is calculated only at virtual sources on the solid circles using a 1D Earth model. For grid points falling in the same interval between two adjacent dashed lines, they share the same Green’s function as that for the central solid line as an approximation, thus saving computation time.

In addition, we present an interval approach to calculate seismograms efficiently given a distance range from a single station. This approximation method can significantly reduce the computational effort for preparing a search database with acceptable accuracy. Figure 2 explains the interval method with a schematic plot. Solid circles with a constant interval are drawn with a seismic station as the centre and any two adjacent dashed lines to the central solid circle mark the zone of an interval. Within every interval after the earth flattening transformation, we calculated the Green’s function for only one virtual source point at the epicentral distance corresponding to the solid circle. For any virtual source at the same depth in the 3D grid, if it falls into the same interval, then it will be assigned the same Green’s function for that interval as an approximation. In this study, our epicentral distance range is from 5° to 15°. If we choose 0.2° as an interval, this means we must calculate seismograms for only 600 (50 intervals × 12 depths) virtual source points, regardless of the number of stations, as opposed to 24,366 (8,122 × 3 stations) virtual sources, reducing the computational effort by a factor of ~40. The calculation of the seismograms to create a database for the Xinjiang test site takes ~13 h on a single workstation. Generating 15,789,168 super-traces and setting up a tree structure for the rapid search takes an additional 30 min. Therefore, creating a search database with synthetics is a very efficient process in practice if high-performance computing resources are available.

### Fast search results

Within the Xinjiang test site, an earthquake with a magnitude of *M*_w_ 5.9 occurred on 8 March 2012 (purple star in Fig. 1). According to the Global Centroid Moment Tensor (CMT) solution on the website^35^, this event was located at 39.49°N, 81.47°E and at a depth of 44.4 km. We use this event as an input and apply our developed earthquake search engine to determine its epicentre and focal mechanism. In addition, we select two other earthquakes to test the system for special situations. These include an earthquake with a magnitude of *M*_w_ 5.3 on 30 April 2014 at 43.02°N, 94.26°E and at a depth of 10.0 km (green star in Fig. 1; outside the test site, according to the NEIC catalogue^36^), and an earthquake with a magnitude of *M*_w_ 5.3 on 15 September 2011 at 36.32°N, 82.50°E and at a depth of 11.6 km (blue star in Fig. 1; inside the test site, according to the NEIC catalogue^36^). For the second additional earthquake on 15 September 2011, we shifted the arrival time of the entire event to simulate it occurring only 40 s after the event on 8 March 2012. Stacking two events at all three stations creates an artificial record with double events. Double events or multiple events are not accounted for in our search database. Therefore, the hypothetic double-event records can be used to test how the search engine would handle the situation.

**Figure 3:**
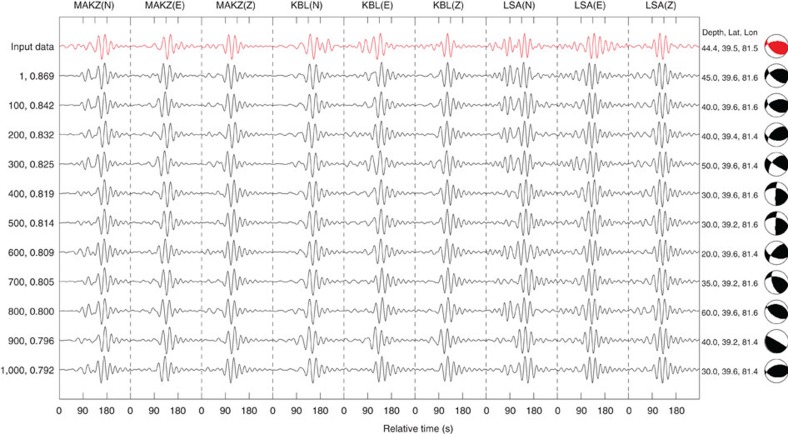
The input data and top 1,000 search results. A comparison is made between the input data from three seismic stations with the Global CMT solution (red) and every 100th result of the top 1,000 search returns (black). The solution sequence index and maximum cross-correlation values are shown on the left. The source depth, latitude, longitude and focal mechanism are shown on the right.

**Figure 4:**
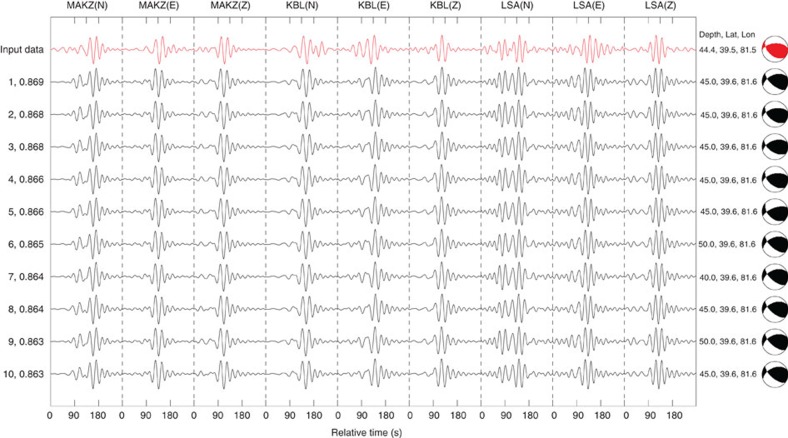
The input data and top 10 search results. A comparison is made between the input data from the three seismic stations with the Global CMT solution (red) and the top 10 search results (black). The solution sequence index and maximum cross-correlation values are on the left. The source depth, latitude, longitude and focal mechanism are on the right.

Figure 3 shows a super-trace of the input entry data for the 8 March 2012 earthquake (in red at the top) and every 100th best match from the database, up to the 1,000th match. Because each super-trace is formed by concatenating three-component data from all three seismic stations, our search is based on fitting all nine full waveforms available from the same event. In Fig. 3, the best search result suggests a source-focal mechanism that is similar to the Global CMT solution, and a source location that is offset from the Global CMT solution by 15 km in horizontal plane and 0.6 km in depth. The top 200 solutions feature their source locations within 25 km and source depths within 5 km of the Global CMT solution. Figure 4 displays the top 10 best matches among the 1,000 solutions. The source-focal mechanisms in all 10 search results are clearly close to the Global CMT solution. Within the best 10 solutions, the maximum cross-correlation value decreases from 0.8695 to 0.8626; thus, their variations are small. This suggests a certain non-uniqueness of the results, which may be due to the limited data and observation geometry. It is important to understand this issue for any solution, because it defines the confidence level for that solution. Regardless, the results indicate that source location, depth and focal mechanism are reasonably well constrained in this case. The source-depth and -focal mechanism estimates seem more reliable than the location estimate in this particular case.

**Figure 5:**
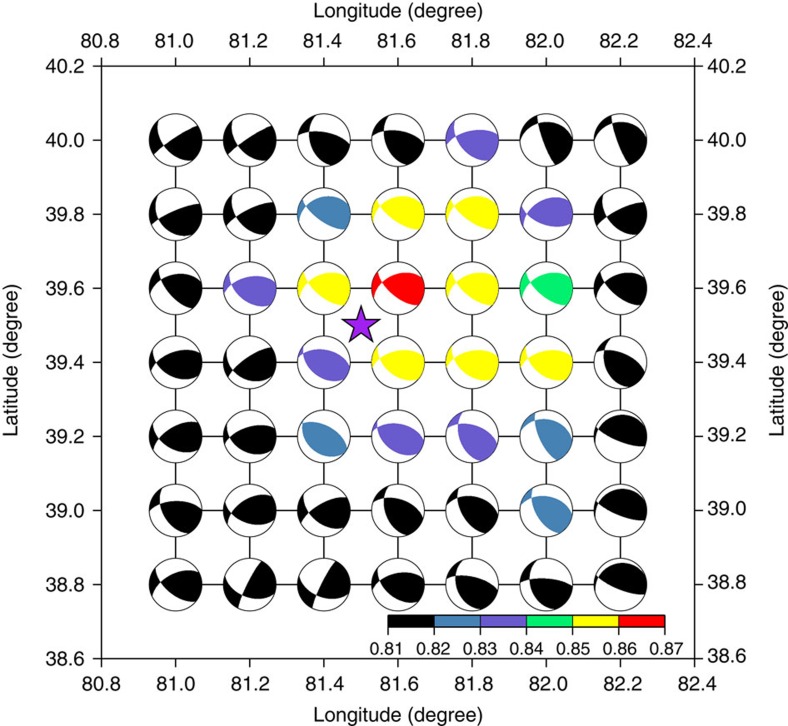
Earthquake lateral location uncertainty. For a source depth at 45 km, the distribution of maximum cross-correlation values between input data and search results, as well as the focal mechanisms around the best match solution, are shown in the beach balls. The purple star marks the event location of the Global CMT solution. The colour bar shows the scale of maximum cross-correlation values.

**Figure 6:**
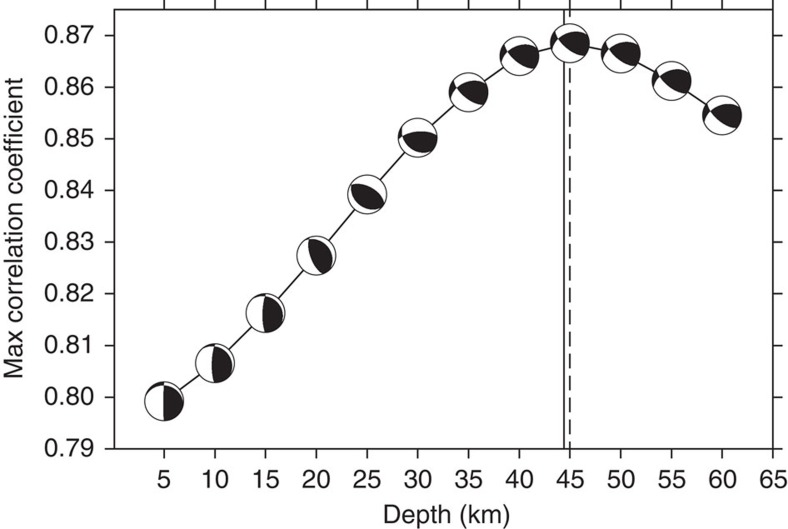
Earthquake depth solution uncertainty. At the surface point of the best match solution, the maximum cross-correlation values between the input data and search results from 5 to 60 km in depth are displayed in the curve. The solid line marks the depth of 44.4 km from the Global CMT solution and the dashed line at 45.0 km shows the best solution from the fast search.

Figure 5 displays the best-match solutions on the test grid at a depth of 45 km along with the Global CMT location of the entry event (purple star). At each grid point, the figure shows focal solution beach balls of the best matches to the entry, along with the maximum cross-correlation coefficients (coloured). Our best search location is ~half a grid interval (0.1°) from the Global CMT location in both latitude and longitude. Plots such as Fig. 5 at multiple depths can help us to understand the solution uncertainty in three dimensions. Figure 6 shows beach balls and maximum cross-correlation coefficients versus depth at the best-match surface location. The dashed line shows that our best estimate for the source depth is 45.0 km, whereas the Global CMT solution reports a depth of 44.4 km. The two solutions are fairly close, which suggests that our result is consistent with the Global CMT solution.

**Figure 7:**
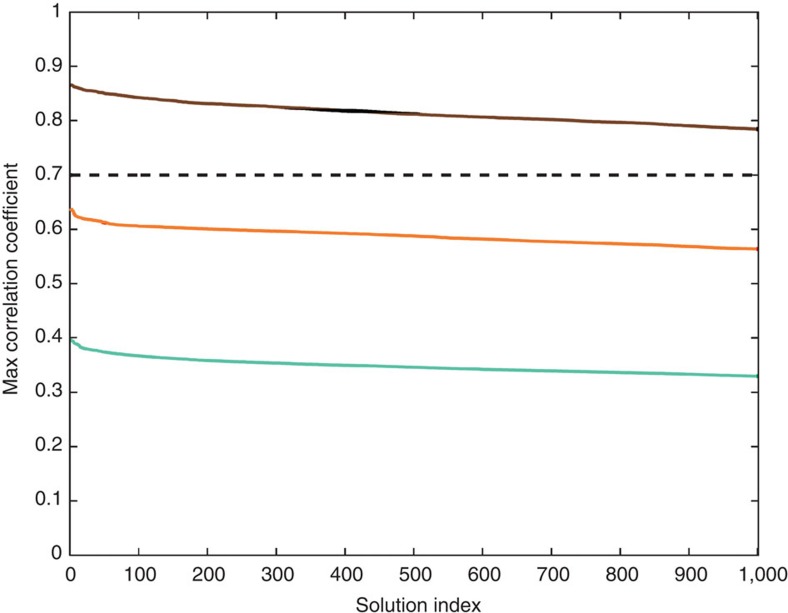
Comparison of search results from testing three earthquakes. The solid curves in the plot present maximum cross-correlation values between the input data and the top 1,000 search results. The brown curve shows the results using an earthquake with a magnitude of *M*_w_ 5.9 that occurred on 8 March 2012 inside the test area. The cyan curve shows the results using an earthquake with a magnitude of *M*_w_ 5.3 on 30 April 2014 outside the test area. The orange curve shows the results using a hypothesised double-event earthquake created from the earthquake on 8 March 2012 and another earthquake with a magnitude of *M*_w_ 5.3 that occurred on 15 September 2011. The dashed line marks the cross-correlation threshold used for validating the search results.

The curve of the maximum cross-correlation coefficient for the best 1,000 search results is shown in brown in Fig. 7. This curve offers a direct indication of the non-uniqueness of the search solutions. If the curve decreases rapidly with the solutions, it suggests that the best solution is well constrained. Otherwise, it indicates that there are too many non-unique or approximate solutions. In this study, the brown curve decreases reasonably rapidly; therefore, the solution confidence is high.

We also used the event on 30 April 2014 outside the test region to test the search engine. The cyan curve in Fig. 7 presents the maximum cross-correlation coefficients for the best 1,000 search results for this event. All of the maximum cross-correlation coefficients are all below 0.40, a significantly small value. This is simply because the events in the search data set cannot match earthquakes outside the area sufficiently well at all three seismic stations. For the same reason, the search of the double-event earthquake entry (described above) also returns low maximum cross-correlation coefficients, as shown by the orange curve in Fig. 7. Through more experiments with synthetic and real data, we found that a cross-correlation coefficient of 0.70 is an effective threshold value for this system to differentiate invalid results in this particular area. These include situations for events outside the data set coverage area, overlapping multiple events and large earthquakes with complex rupture process. This suggests that the earthquake search engine could validate the results automatically and avoid false reporting. When the maximum cross-correlation coefficient between an entry and its search results is below 0.70 in these test cases, the search results are invalid. These results suggest that either the current search database should be updated or alternative methods are needed to process the data. For different areas and monitoring networks, such threshold values must be estimated and set before applying the system. If the search database could include special events, the search engine approach should be able to handle the situations. We shall discuss several possibilities to improve the database.

## Discussion

We have demonstrated through the Xinjiang test site that by applying the earthquake search engine, we are able to report an earthquake’s location, depth and source-focal mechanism in <1 s after receiving the long-period surface wave data. Instead of one solution, the search engine actually returns a subspace of approximate solutions that can help us to assess the solution confidence within a second as well. This significant improvement in the reporting time of source-focal mechanisms could help to issue tsunami warnings and monitor major fault activities more effectively. In the search process, we did not discuss the source parameter of the earthquake magnitude. This is because event magnitude can be estimated instantly using amplitude information^37^.

This new approach requires creating a search database for the area of interest at a local or regional monitoring distance. Our forward modelling programme is limited to a point source; therefore, this study targets earthquakes with magnitude *M*_w_ <7.5. However, our search engine is not inherently limited to specific source sizes or wave types. If we can create a database that includes simulated large earthquakes, then the search engine would be able to find best waveform matches and help obtain earthquake parameters. The challenge for creating such a search database is the complexity in the dynamic rupture process of a large earthquake. Possible solutions could include properly weighting intermediate- and low-frequency data considering the source size^2^, calculating synthetics with quasi-finite source Green’s functions^12^ and defining a centroid location from the search results with the largest variance reduction^15^. Some earthquakes do repeat in history. It will also be interesting to establish a search database using historic events with known source information for seismically active areas.

Our case study presents input data dominated by low-frequency surface waves. For a local or regional area with relatively accurate three-dimensional velocity models available, such as southern California, the earthquake search engine presented in this study could use high-frequency body waves. In this case, because the Green’s functions for the body waves can be reasonably estimated, the earthquake search engine could be used to rapidly report source-focal mechanisms (in addition to the location and magnitude) using the first arrival P waves within a second after receiving the data. For broad-band or high-frequency data, aligning the P-wave arrival of the input data with synthetics in the database is a concern. Fortunately, it is easy to align noise-free synthetic seismograms in the database. For the input data, we simply apply a series of small positive and negative shifts to the P-wave arrival and simultaneously conduct a fast search with all the shifted entries, along with the original entry. We pick the results associated with the smallest misfit for the final solutions. This method helps remove the effect of an incorrect P-wave arrival.

In this study, we select an area with a size of 5° by 5° and a source depth from 5 to 60 km in Xinjiang, China and test our earthquake search engine on a single CPU computer. If we increase the size of the interest zone in any dimension or decrease the gridding intervals for virtual source locations and focal solutions, the search database will increase as well. Our interval approach for calculating Green’s functions suggests that the computation time for the synthetic data set is only associated with distance between the hypocentre and a seismic station; thus, having more seismic stations or networks involved does not significantly increase the computation time. Considering the profound and unique data information associated with body waves and surface waves at different frequencies, we could design multiple parallel search processes with different data information from the same earthquake to ensure reliable results. We may also be able to apply the search engine approach to streaming waveform data in real time and keep updating the results as we receive more data from the same earthquake. These ideas warrant further study.

## Methods

### Computer fast search technology

The earthquake search engine presented in this study uses fast search methods that are similar to those that have been applied in web search engines, which are designed primarily to find text-based information on the web, and the bulk of the search effort is in indexing and ranking a large database^20^. An image search engine is similar but is based on image content, in which the content similarity assessment, along with other supplementary information, is used to retrieve the best matches to an entry image^21^,^22^,^23^. Internet technologies have also inspired research in other areas. For example, Aguiar and Beroza^38^ applied an Internet ranking method to analyse earthquake waveform similarity.

In the computer search field, the number of sampling points in data is regarded as its ‘dimension.’ A seismogram similarity search is a high-dimensional problem that requires a substantial effort to search for a true solution from a large data set. Dimension-reduction methods can reduce the problem size by decreasing the number of time samples necessary to represent the data while maintaining the essential data characteristics^39^. In addition to dimension reduction, an important approach is the development of approximate nearest-neighbour search methods that can be orders of magnitude faster than the exact search, while still providing near-optimal accuracy^24^.

Existing fast search methods are either hash- or tree-based, such as Locality-Sensitive Hashing^25^ and the multiple randomized K-dimensional (MRKD) tree method^26^,^27^. After comparing the two approaches for dealing with seismograms, we found that the MRKD-tree method is consistently faster for various sizes of high-dimensional seismogram data sets. Both Silpa-Anan and Hartley^26^ and Muja and Lowe^24^ reported that the MRKD-tree method provides the best performance in computer vision problems for processing high-dimensional data as well. Therefore, we shall focus our effort on the MRKD-tree method in this study. This method involves creating multiple tree structures from data sets by splitting the waveform data in half at each level of the tree for the time sample in which the data exhibit the greatest variance in amplitude. The database captures the most prominent characteristics in the earthquake data with limited samples. We then search the best matches following the tree structure when an entry arrives. The method requires log_2_*N* comparisons to find the first candidate for the best match from one tree, where *N* is the number of seismograms in the database^28^. Additional backtracking effort is needed if more search returns are required.

### Indexing and ranking seismograms by MRKD trees

**Figure 8:**
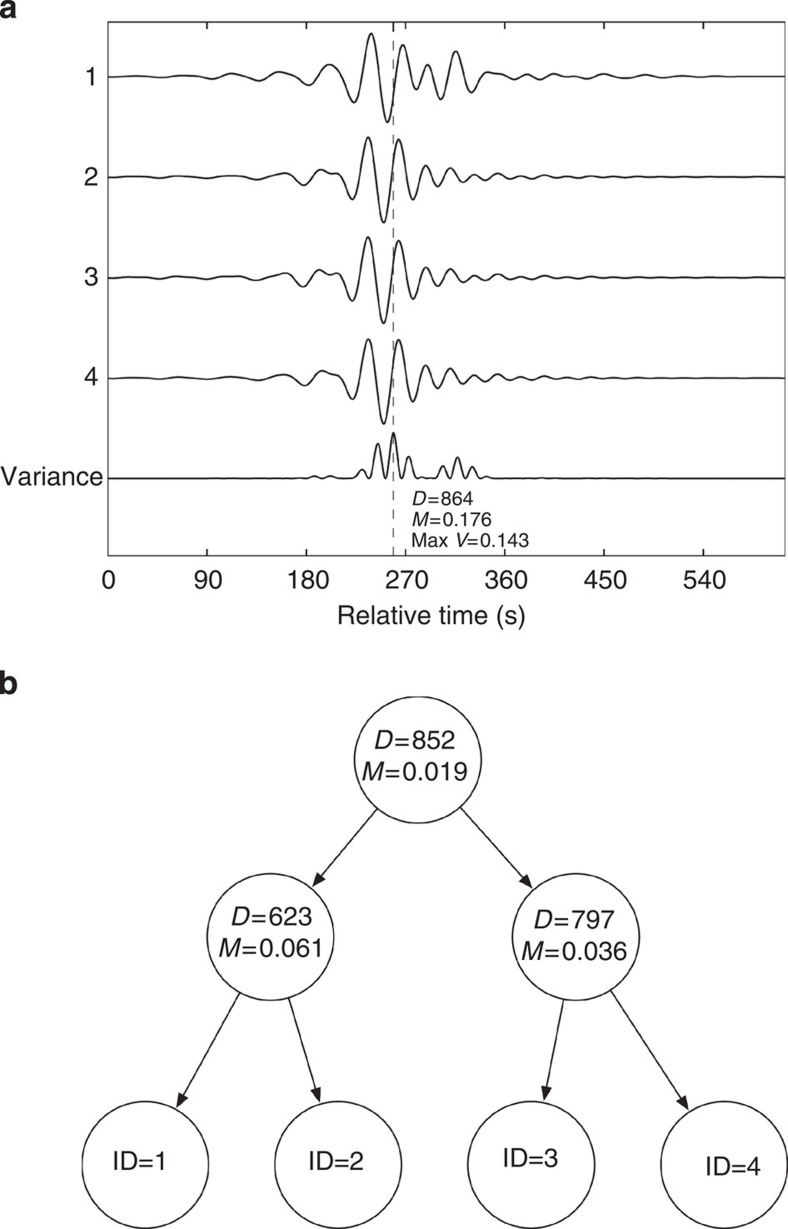
Indexing earthquake waveforms for fast search. A MRKD tree method is applied to create a database tree for fast search. (**a**) Assuming a data set of four seismograms, the mean and RMS variance for each data dimension are calculated for all of the traces; (**b**) At the top of the tree, the largest variance (0.143) occurs at the sample 864 and the seismograms with amplitudes at this sample less than the mean (0.176) are placed on the left, those larger than the mean are placed on the right. The process is recursively applied at each level until only one seismogram remains on each side.

Figure 8 illustrates how a K-dimensional tree is created with an example of four seismograms assumed in the database. The first arrivals of the four seismograms are picked and aligned. At each time sample (dimension), the amplitude mean across four seismograms is calculated. The bottom curve in Fig. 8a shows the amplitude variance relative to the mean at each time sample. The largest amplitude variance (0.143) relative to the mean (0.176) is found at time sample 864, which is selected to start a tree. At the same selected time sample, seismograms with amplitudes lower than the mean (ID=1, 4) are placed on the left of a dimension node, while those with amplitudes greater than the mean (ID=2, 3) are placed on the right. The above process is recursively applied to seismograms on the left and right separately, until only one seismogram (leaf node) remains below any dimension node.

Figure 8b shows a two-level tree established following the above process. In reality, multiple randomised K-D trees should be created from the same data set for the search. To create multiple trees, we follow the approach by Muja and Lowe^24^ and choose another split dimension randomly from the first *m* dimensions in which the data have the greatest variance. In our applications, we used the fixed value *m=5* and created 128 trees from a large earthquake data set. When a query seismogram comes in, its amplitude variance relative to the mean of the data set at the same dimension (time sampling point) will be compared with the partitioning value to determine to which half of the data the query data belong. This searching process is performed among all the trees separately. At each dimension node, an accumulated L_2_ distance between the input amplitude value and the mean of the data set is calculated, and a single priority queue across all the randomised trees is maintained by increasing the L_2_ distance. At each leaf node where a seismogram ID is available on the tree, the L_2_ norm distance between the entry and the synthetic seismogram is also calculated and placed in a candidate queue with increasing distance. It requires an iterative search to find multiple approximate solutions. The priority queue determines the seismogram to be picked for comparison in the next iteration, and the candidate queue returns a number of results in the order of decreasing similarity to the entry seismogram^26^. The idea behind this search approach is to settle for an approximate nearest neighbour to an entry with the highest probability, and it may not always return the exact nearest neighbours. That principle is acceptable in seismogram matching because an exact match to a real seismogram essentially does not exist due to our incomplete knowledge of the 3D Earth’s structure and limited ability to accurately reproduce real wave-propagation effects.

### Optimising fast search performance

Dimension reduction is another important effort to further speed up the search process. Its goal is to identify patterns in the data and express the data in a compact form to highlight their similarities and differences. Among dimension-reduction methods, principal components analysis (PCA) has proved to be reliable for embedding the data into a linear subspace of lower dimensionality^39^. To apply PCA, we first calculate the covariance matrix for the data set and then solve its eigenvectors and eigenvalues. For a data set with *n* dimensions, there should be *n* eigenvectors and eigenvalues. If we choose only the first *p* eigenvectors associated with the top *p* large eigenvalues, then the final data set has only *p* dimensions, without losing much information. In practice, we need to examine the eigenvalue distribution and determine an optimal value for *p*.

**Figure 9:**
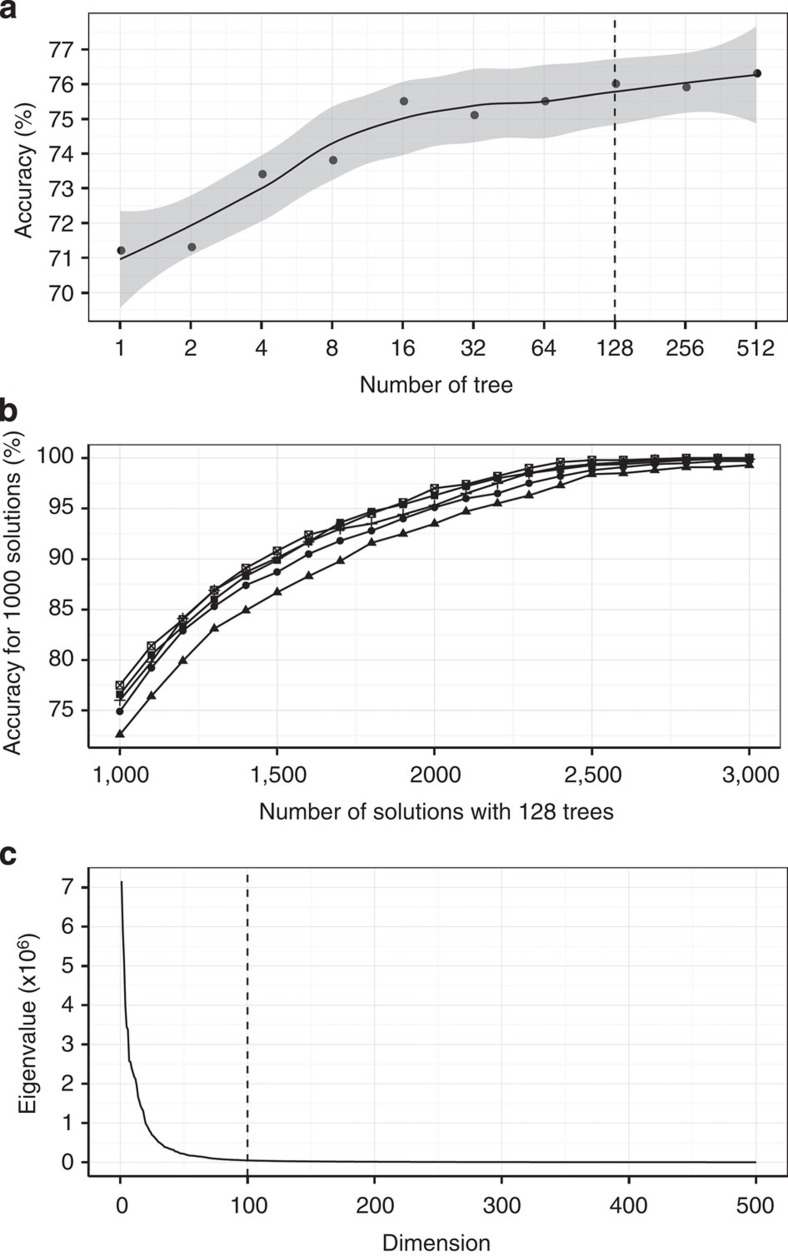
Estimating the optimal parameters for fast search. Accuracy is defined as the percentage of the number of identical solutions between MRKD tree and an exact search over 1,000 search results. The dashed line marks the parameter selected. (**a**) For 1,000 search results, the search accuracy versus the number of trees is presented. In total, 128 trees are selected to produce accuracy of ~76%. (**b**) With 128 trees, the search accuracy for the top 1,000 solutions versus the total number of output solutions for five random synthetic entries is shown. A total of 1,000 output solutions are sufficient to produce an accuracy of ~76%. (**c**) To apply PCA dimension reduction, the plot of eigenvalues of the data covariance matrix versus dimensions is presented. Those eigenvalues close to zero at dimension above 100 can be removed and the resulting new data set has only 100 dimensions without losing much information.

It is important to select optimal parameters for applying a fast search method. Figure 9 illustrates how we select the number of trees, the number of search results and the PCA output dimensions for dealing with over 15 million synthetic seismograms in a data set. Figure 9a shows a plot of the search accuracy versus the number of trees applied in the MRKD-tree method for returning 1,000 results. The search accuracy is defined as the ratio of identical solutions between the MRKD tree and exact search over the total 1,000 search results. For 128 trees, its accuracy is ~76%, which means that out of the given 1,000 results, 760 fast search solutions are identical to those from the exact search. This accuracy should be sufficient to select the best waveform matches. Figure 9b presents a plot of the search accuracy for the top 1,000 solutions versus the number of search solution candidates based on the testing of five different synthetic earthquake entries. More search returns yield more accurate results among the top 1,000 solutions, but it requires more computation. Selecting 1,000 solutions and 128 trees seem to reach a good balance between accuracy and computational effort. For the above testing data, an original trace consists of 3,072 time samples (dimensions). Figure 9c displays a plot of the eigenvalues versus the dimensions derived from the data covariance matrix. This plot is needed to determine the optimal *p* for applying the PCA dimension reduction discussed above. The plot suggests that the eigenvalues at dimensions above 100 are close to zero. Thus, those eigenvectors associated with small eigenvalues can be removed without losing much information. An optimal *p* of 100 is selected and the dimensions of the new data set are reduced to 100.

**Figure 10:**
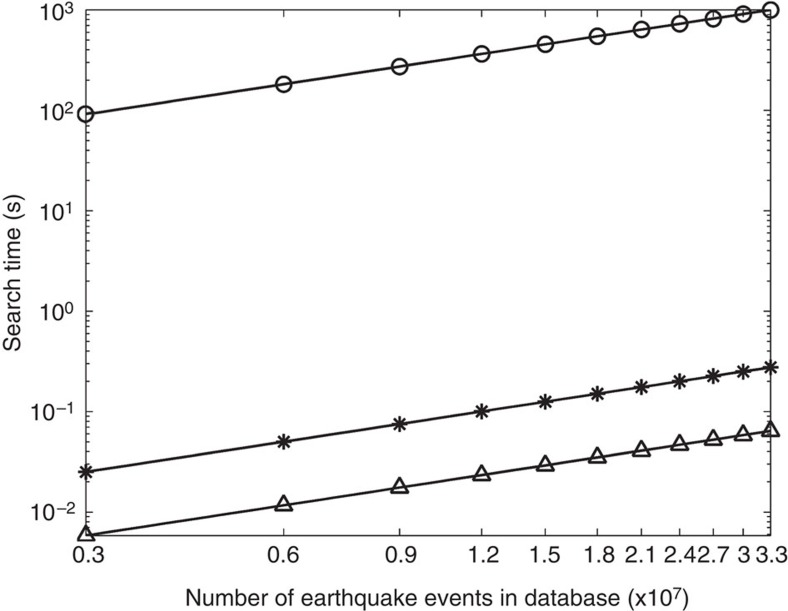
Comparison of computer search time for different methods. Three different methods are tested for returning 1,000 best match results on a single CPU. The methods include the exact linear search (open circle), the MRKD tree method without dimension reduction (star) and the MRKD-tree method with PCA applied for dimension reduction (open triangle).

The performance of computer search algorithms varies widely based on properties of the data sets^24^. For the three-component seismic stations, we first normalise the waveforms and then merge the three-component data to create a super-trace from each event. Figure 10 shows that the MRKD-tree method can find 1,000 best matches to the entry among 33 million super-traces in ~0.2 s without PCA and in ~0.06 s with PCA. An exact search to calculate the L_2_ norm of the misfit takes over 17 min to accomplish the same job. Applying cross-correlation to find best matches takes over 27 h. These tests are performed on a single AMD Opteron processor 6136. As mentioned above, the MRKD-tree method requires log_2_*N* comparisons to find the first candidate for the best match on a single tree, where *N* is the number of seismograms in the database^28^. However, in Fig. 10 the curves of MRKD-tree search time versus the number of earthquakes are nearly linear in the log–log plot; this is because in practice we split the data set into multiple smaller data sets for separate sequential searches and then merge the results. The data sets are structured for parallel implementation in the future.

## Author contributions

J.Z. designed the project, analysed the data and wrote the manuscript. H.J.Z. and E.H.C. also analysed the results and helped in writing the manuscript. Y.Z. and X.Z. tested the method. Y.Z. and W.H.K. processed and analysed the data.

## Additional information

**How to cite this article:** Zhang, J. *et al.* Real-time earthquake monitoring using a search engine method. *Nat. Commun.* 5:5664 doi: 10.1038/ncomms6664 (2014).
